# Supplementary material for: Transgenic tools for targeted chromosome rearrangements allow construction of balancer chromosomes in non-melanogaster Drosophila species
Source: G3 (Bethesda). 2022 Feb 10;12(4):jkac030. doi: 10.1093/g3journal/jkac030 (PMC8982376; doi:10.1093/g3journal/jkac030)
Supplement: jkac030_Supplementary_Data [file jkac030_supplementary_data.docx]

SUPPLEMENTARY INFORMATION

Supplementary Material – All plasmids used in this study were re-sequenced in their entirety and annotated sequences are provided.

Table S1 – gBlocks used to generate new plasmids.

| gBlock name | Sequence |
| --- | --- |
| 3Xtin-prom | CGATCATGCGCACCCGTGGCCAGGGCCGCACATATGCAATGGTTAATTCGAGCTCGCCCGGGGCTCTCAAGTGTGCACTCAAGTGTGCTCTCAAGTGTGATCCAAGCTTATCGATTTCGAACCCTCGACCGCCGGAGTATAAATAGAGGCGCTTCGTCTACGGAGCGACAATTCAATTCAAACAAGCAAAGTGAACACGTCGCTAAGCGAAAGCTAAGCAAATAAACAAGCGCAGCTGAACAAGCTAAACAATCTGCAGTAAAGTGCAAGTTAAAGTGAATCAATTAAAAGTAACCAGCAACCAAGTAAATCAACTGCAACTACTGAAATCTGCCAAGAAGTAATTATTGAATACAAGAAGAGAACTCTGAATAGATCTAAAAGGTAGGTTCAACCACTGATGCCTAGGCACACCGAAACGACTAACCCTAATTCTTATCCTTTACTTCAGGCGGCCGCGGCTCGAGGGTACCAATCAAACATGTCCAAAGGTGAAGAACTGTTTACCGG |
| 9xtin-prom | CGATCATGCGCACCCGTGGCCAGGGCCGCACATATGCAATGGTTAATTCGAGCTCGCCCGGGGCTCTCAAGTGTATCTGAGACTCAAGTGTGCTCACTTGAGTGCTCTCAAGTGTATGACCACACTTGAGTGCTCTCAAGTGTTCGATCTCTCAAGTGTACTTCACTCAAGTGTATGATGCTCTCAAGTGTATCCAAGCTTATCGATTTCGAACCCTCGACCGCCGGAGTATAAATAGAGGCGCTTCGTCTACGGAGCGACAATTCAATTCAAACAAGCAAAGTGAACACGTCGCTAAGCGAAAGCTAAGCAAATAAACAAGCGCAGCTGAACAAGCTAAACAATCTGCAGTAAAGTGCAAGTTAAAGTGAATCAATTAAAAGTAACCAGCAACCAAGTAAATCAACTGCAACTACTGAAATCTGCCAAGAAGTAATTATTGAATACAAGAAGAGAACTCTGAATAGATCTAAAAGGTAGGTTCAACCACTGATGCCTAGGCACACCGAAACGACTAACCCTAATTCTTATCCTTTACTTCAGGCGGCCGCGGCTCGAGGGTACCAATCAAACATGTCCAAAGGTGAAGAACTGTTTACCGG |
| 3xmef2-prom | CGATCATGCGCACCCGTGGCCAGGGCCGCACATATGCAATGGTTAATTCGAGCTCGCCCGGGGCACGGCTAAAAATAGCGTTGTATAGGCTAAAAATAGCCTGACGTAAGCTAAAAATAGCGTTATCCAAGCTTATCGATTTCGAACCCTCGACCGCCGGAGTATAAATAGAGGCGCTTCGTCTACGGAGCGACAATTCAATTCAAACAAGCAAAGTGAACACGTCGCTAAGCGAAAGCTAAGCAAATAAACAAGCGCAGCTGAACAAGCTAAACAATCTGCAGTAAAGTGCAAGTTAAAGTGAATCAATTAAAAGTAACCAGCAACCAAGTAAATCAACTGCAACTACTGAAATCTGCCAAGAAGTAATTATTGAATACAAGAAGAGAACTCTGAATAGATCTAAAAGGTAGGTTCAACCACTGATGCCTAGGCACACCGAAACGACTAACCCTAATTCTTATCCTTTACTTCAGGCGGCCGCGGCTCGAGGGTACCAATCAAACATGTCCAAAGGTGAAGAACTGTTTACCGG |
| 9xmef2-prom | CGATCATGCGCACCCGTGGCCAGGGCCGCACATATGCAATGGTTAATTCGAGCTCGCCCGGGGCACGGCTAAAAATAGCGTTGCTGAATTCTGAATTATAGGCTAAAAATAGCCTGACGTTCTAATCGTAAGCTAAAAATAGCGTTATATTTCCATAGTCGGATGGCACGGCTAAAAATAGCGTTGCGAAACTGCTTATATAGGCTAAAAATAGCCTGATTTACGGACGTAAGCTAAAAATAGCGTTATTTACTACGGACTGGCACGGCTAAAAATAGCGTTGAATGTCTAAATTATATAGGCTAAAAATAGCCTGAAATTTAACTAGACTCTACGTAAGCTAAAAATAGCGTTATCCAAGCTTATCGATTTCGAACCCTCGACCGCCGGAGTATAAATAGAGGCGCTTCGTCTACGGAGCGACAATTCAATTCAAACAAGCAAAGTGAACACGTCGCTAAGCGAAAGCTAAGCAAATAAACAAGCGCAGCTGAACAAGCTAAACAATCTGCAGTAAAGTGCAAGTTAAAGTGAATCAATTAAAAGTAACCAGCAACCAAGTAAATCAACTGCAACTACTGAAATCTGCCAAGAAGTAATTATTGAATACAAGAAGAGAACTCTGAATAGATCTAAAAGGTAGGTTCAACCACTGATGCCTAGGCACACCGAAACGACTAACCCTAATTCTTATCCTTTACTTCAGGCGGCCGCGGCTCGAGGGTACCAATCAAACATGTCCAAAGGTGAAGAACTGTTTACCGG |
| GAGA-3Xtin | GGGTCCTCAACGACAGGAGCACGATCATGCAGAGAGTCTCTCAAGTGTGTAGAGAGTCACTCAAGTGTGACTCTCTACTCTCAAGTGATTAGAGATATCGATTTCGAACCCTCGACCGCCGGAGT |
| GAGA-3XM2 | AATTGGAGCTCCACCGCGGTGGCCATTAATTAACGCCCGGGGAGAGAGTACACGGCTAAAAATAGCGTTGCTAGAGAGCATATAGGCTAAAAATAGCCTGAATCTCTCTGACGTAAGCTAAAAATAGCGTTATCAGAGAGCAAGCTTAATTAACCCTCGACCGCCGGAGTATAAATAG |
| Act88F-145>GFP-GW | AATTGGAGCTCCACCGCGGTGGCCATTAATTAAGGATGCAGCTTCCTCGAGCACCGCGCGCGGAGATCTCGATCAACCTTGATGTTGATTTATAGGTGCCGCTCTGCTTGGCGCGTCTATTTTAGATTCGCCTCGCTGCGTGCCCGTTGAAATGTCCCATTCTCCCAGTCCCTGCCGCTTAATTAACCCTCGACCGCCGGAGTATAAATAG |
| Act88F-285>GFP-GW | AATTGGAGCTCCACCGCGGTGGCCATTAATTAAGCTGGCAATGGTTGGTTAATTGCACTGATAAATGGTCGGCACGGTGATTTCGCATCTTCGGGATTGCATCGGCGCCGCAATGCAAAGTGCAGCAGCATTCTGTAGAATGCGATTGCAAATGTGGATGCAGCTTCCTCGAGCACCGCGCGCGGAGATCTCGATCAACCTTGATGTTGATTTATAGGTGCCGCTCTGCTTGGCGCGTCTATTTTAGATTCGCCTCGCTGCGTGCCCGTTGAAATGTCCCATTCTCCCAGTCCCTGCCGCGGATGCCAATTGTCTTGCTTAATTAACCCTCGACCGCCGGAGTATAAATAG |
| Mhc_F4-453-promoter | CGATCATGCGCACCCGTGGCCAGGGCCGCACATATGCAATGACGTAACAAATACCAACGCCCCGAAAATCCGTATATATGCAAAGCATTCTGATATATATATATATAAGTGGTATACATATACATATTCGTATCGGATCCGTGATCTATATTTAGGCGACAACCCACACAATTCACTTGTTGTCTGTTTATAACAAAAACAACAACACAAGCACCGACCAGGCCTAATTAACTTTGAGGTGTGAGATAAAATCAAAGCCACACATAAATAAATATCCGGTTACGGTAACGGGAATTTCGAAAGCGCGAAACGGGAAAAATGTTCATATTTGGAAATCGAAATTAAATTTCGAAATCCTATCCACGCACACACGCGTAGAACTGTTATAGTGTCCGCTATTGCTGCTGCTGTCAAAATGGGTCAAAAGCATAAAATCCATAAAAGAATCAGACTTCGAAACAAAATTCCAAAAATAGTATGCTTTTCTGAGAACCATCGATTTCGAACCCTCGACCGCCGGAGTATAAATAGAGGCGCTTCGTCTACGGAGCGACAATTCAATTCAAACAAGCAAAGTGAACACGTCGCTAAGCGAAAGCTAAGCAAATAAACAAGCGCAGCTGAACAAGCTAAACAATCTGCAGTAAAGTGCAAGTTAAAGTGAATCAATTAAAAGTAACCAGCAACCAAGTAAATCAACTGCAACTACTGAAATCTGCCAAGAAGTAATTATTGAATACAAGAAGAGAACTCTGAATAGATCTAAAAGGTAGGTTCAACCACTGATGCCTAGGCACACCGAAACGACTAACCCTAATTCTTATCCTTTACTTCAGGCGGCCGCGGCTCGAGGGTACCAATCAAACATGTCCAAAGGTGAAGAACTGTTTACCGG |
| Mhc_F4-678-promoter | CGATCATGCGCACCCGTGGCCAGGGCCGCACATATGCAATGATACTCGAATTCGTTGTCGGCTCATATACATGGGCGAAATAATTTCGAATATGTTTTAAAAATAACCAAAGACATTAGAAAGAGATCGCCAATACTTATACATTATGTCTATGTGTGCCATGTGGTAGCATGAGCCAAAAAGCTTCTCGAAATTACGAATTACATATAGACGAATATGTATGTGACTTTAGTTTCGAAATAATTTCGAGAATTTTAAAAATAACGCATTCGTTAAAAGTTCGCGTCAATTCGAATCGAATTTTCGATTGTCGATTTAGTGTGGATTGTCGAAAATCGTTCCGCCTTCGAAGTTTACTGAAAGGAATCATTGCGATCTCGTGAATTGCTTGTATGAGAACACGCCACCATATCGAGATACCTGTTCAAAATATCGAAATCGCGTTCCAAACGGTAGTAGTTACCAGTTGAGTGAGTTGTGGTGACAGTTTAACCTCTCTACATTTGTAATAATAAGCATTGTTTTGGCGTATCAATAGTCTGTAGATTTTTTCACAAAGAAACCTGGAGAAATGTACACAAATATCTAAATGAAAATTGGCGTTACGTAACAAATACCAACGCCCCGAAAATCCGTATATATGCAAAGCATTCTGATATATATATATATAAGTGGTATACATATACATATTCGTATCGGATCCGTGATCTATATTTAGGATCGATTTCGAACCCTCGACCGCCGGAGTATAAATAGAGGCGCTTCGTCTACGGAGCGACAATTCAATTCAAACAAGCAAAGTGAACACGTCGCTAAGCGAAAGCTAAGCAAATAAACAAGCGCAGCTGAACAAGCTAAACAATCTGCAGTAAAGTGCAAGTTAAAGTGAATCAATTAAAAGTAACCAGCAACCAAGTAAATCAACTGCAACTACTGAAATCTGCCAAGAAGTAATTATTGAATACAAGAAGAGAACTCTGAATAGATCTAAAAGGTAGGTTCAACCACTGATGCCTAGGCACACCGAAACGACTAACCCTAATTCTTATCCTTTACTTCAGGCGGCCGCGGCTCGAGGGTACCAATCAAACATGTCCAAAGGTGAAGAACTGTTTACCGG |
|  |  |

Table S2. Plasmids generated in this study. All plasmids are available from AddGene.

| Plasmid Name | AddGene ID |
| --- | --- |
| pENTR{FRT} | 182163 |
| pENTR{FRT-RC} | 182164 |
| pENTR{KDRT} | 182165 |
| pENTR{KDRT-RC} | 182166 |
| p{3XP3-eGFP::GW,attB} | 182167 |
| p{3XP3-dsRed::GW,attB} | 182168 |
| p{3XP3-mTurquoise::GW,attB} | 182169 |
| p{Mhc-F4-678-EGFP::GW,attB} | 182170 |
| p{Mhc-F4-678-DsRed::GW,attB} | 182171 |
| p{ie1-eGFP::GW,attB} | 182172 |
| p{ie1-DsRed::GW,attB} | 182173 |
| p{ie1-eGFP::FRT,attB} | 182174 |
| p{ie1-eGFP::FRT-RC,attB} | 182175 |
| p{3XP3-dsRed::FRT,attB} | 182176 |
| p{3XP3-dsRed::FRT-RC,attB} | 182177 |
| p{3XP3-dsRed::KD,attB} | 182178 |
| p{3XP3-dsRed::KD-RC,attB} | 182179 |
| p{3XP3-mTurquoise::FRT,attB} | 182180 |
| p{3XP3-mTurquoise::FRT-RC,attB} | 182181 |
| p{3XP3-mTurquoise::KD,attB} | 182182 |
| p{Mhc-F4-678-eGFP::KD,attB} | 182183 |
| p{Mhc-F4-678-GFP::KD-RC,attB} | 182184 |
| p{Mhc-F4-678-eGFP::FRT,attB} | 182185 |
| p{Mhc-F4-678-DsRed::FRT,attB} | 182186 |
| p{Mhc-F4-678-DsRed::FRT-RC,attB} | 182187 |
| p{Mhc-F4-678-DsRed::KD,attB} | 182188 |
| p{Mhc-F4-678-DsRed::KD-RC,attB} | 182189 |
| p{ie1-DsRed::FRT,attB} | 182190 |
| p{ie1-DsRed::FRT-RC,attB} | 182191 |
| T7-phiC31-nos3'UTR | 182192 |
| pBac{3Xtin-eGFP,w+,attB} | 182193 |
| pBac{9Xtin-eGFP,w+,attB} | 182194 |
| pBac{3Xtin-GAGA-eGFP,w+,attB} | 182195 |
| pBac{3Xmef2-eGFP,attB,w+} | 182196 |
| pBac{9Xmef2-eGFP,attB,w+} | 182197 |
| pBac{Act88F-eGFP,attB,w+} | 182198 |
| pBac{Mhc-453-GFP,attB,w+} | 182199 |


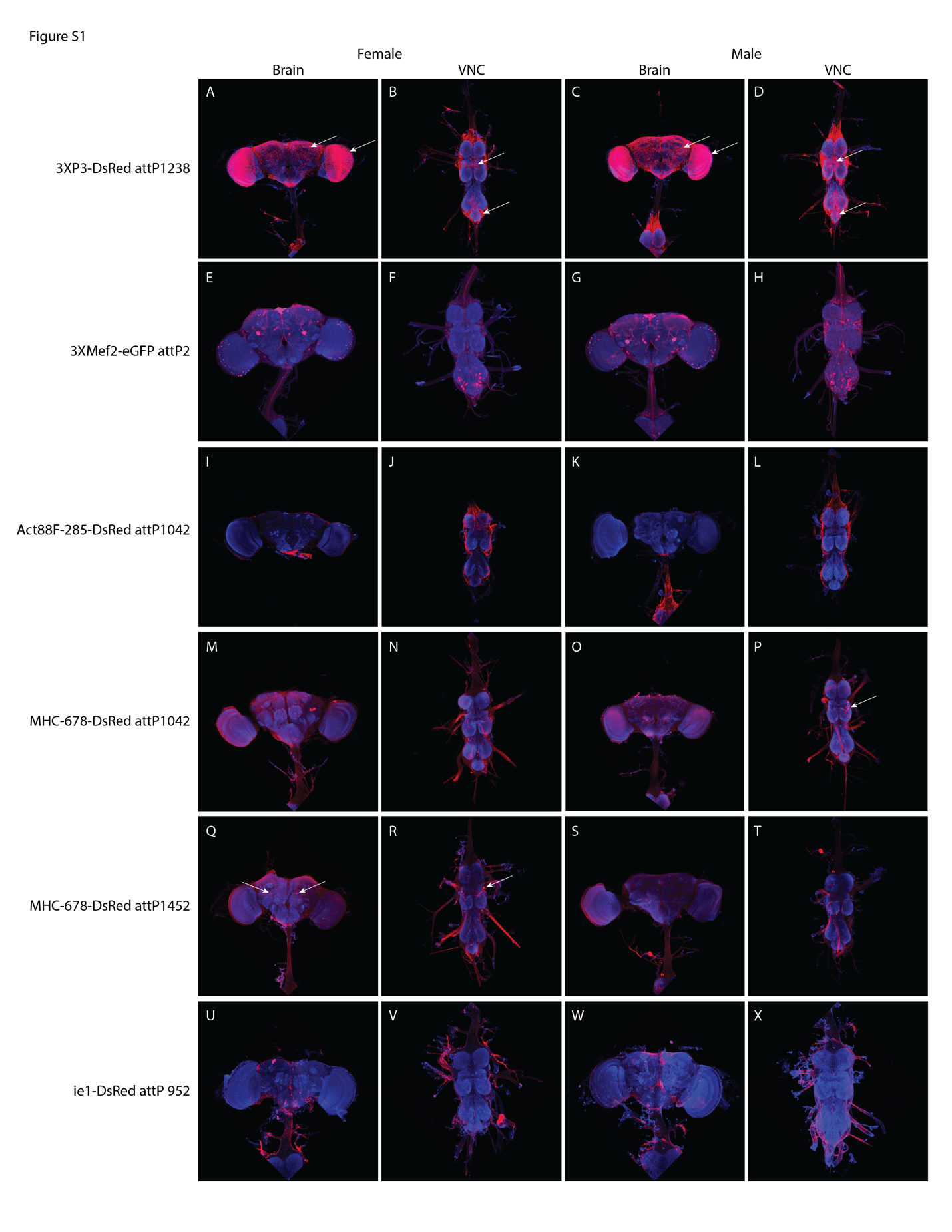


Figure S1 – Expression patterns of fluorescent reporter genes in nervous system. Brains (columns 1 and 3) and ventral nerve cords (VNC; columns 2 and 4) from females (columns 1 and 2) and males (columns 3 and 4) stained for reporter genes (red) and neuropil (blue).

A-D – Enhancer *3XP3* drives strong expression in optic lobe, as expected, but also in many other regions of the brain and VNC (white arrows point to regions of neuronal expression).

E-H –Enhancer *3XMef2* drives expression in some neurons of the brain and ventral nerve cord and what appear to be glia.

I-L – The *Act88F-285* enhancer did not drive detectable expression in brain or VNC cells. Red in these images is autofluorescence from non-neuronal tissue.

M-T – The *MHC-687* enhancer in two different landing sites drove stochastic expression in a few neurons, most obviously a motor neuron of the mesothoracic segment.

U-X – The *ie1* enhancer does not drive detectable expression in brain or VNC.


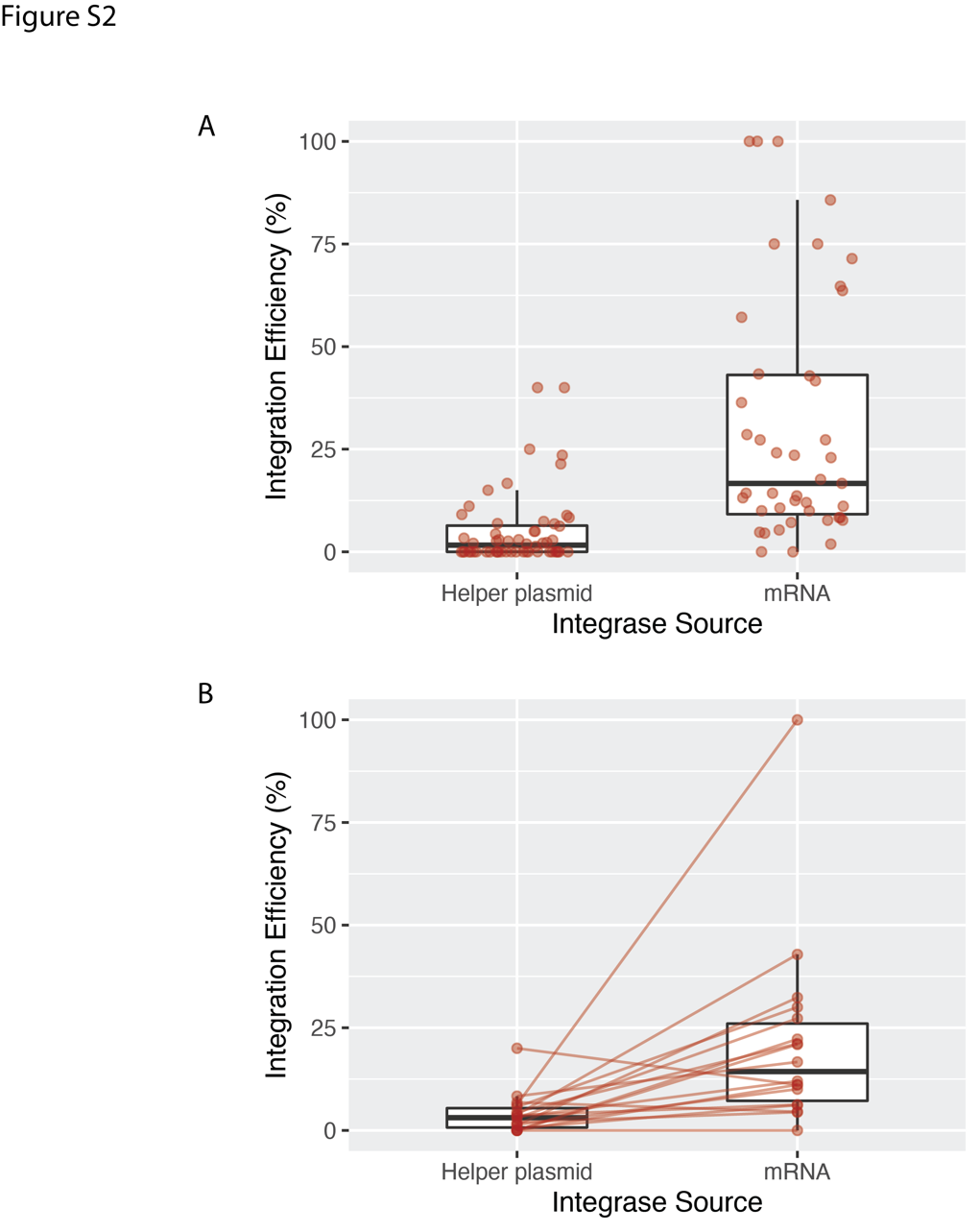


Figure S2 – *In vitro* transcribed mRNA for phiC31 integrase provides increased integration efficiency compared with use of helper plasmid. Embryos carrying *attP* sites were injected with many different *attB* plasmids and co-injected with either pBS130 (56 injections), a “helper” plasmid carrying a heat-shock inducible *phiC31 integrase* gene*,* or *in vitro* transcribed *phiC31 integrase* mRNA. (A) Values indicate the proportion of fertile G0 animals that yielded offspring with integration events for all injections. (B) Injections of either helper plasmid of mRNA for integration of an *attB* plasmid for the subset of injections that were targeted to the same landing site. Red lines connect data for different injections into the same *attP* landing site.


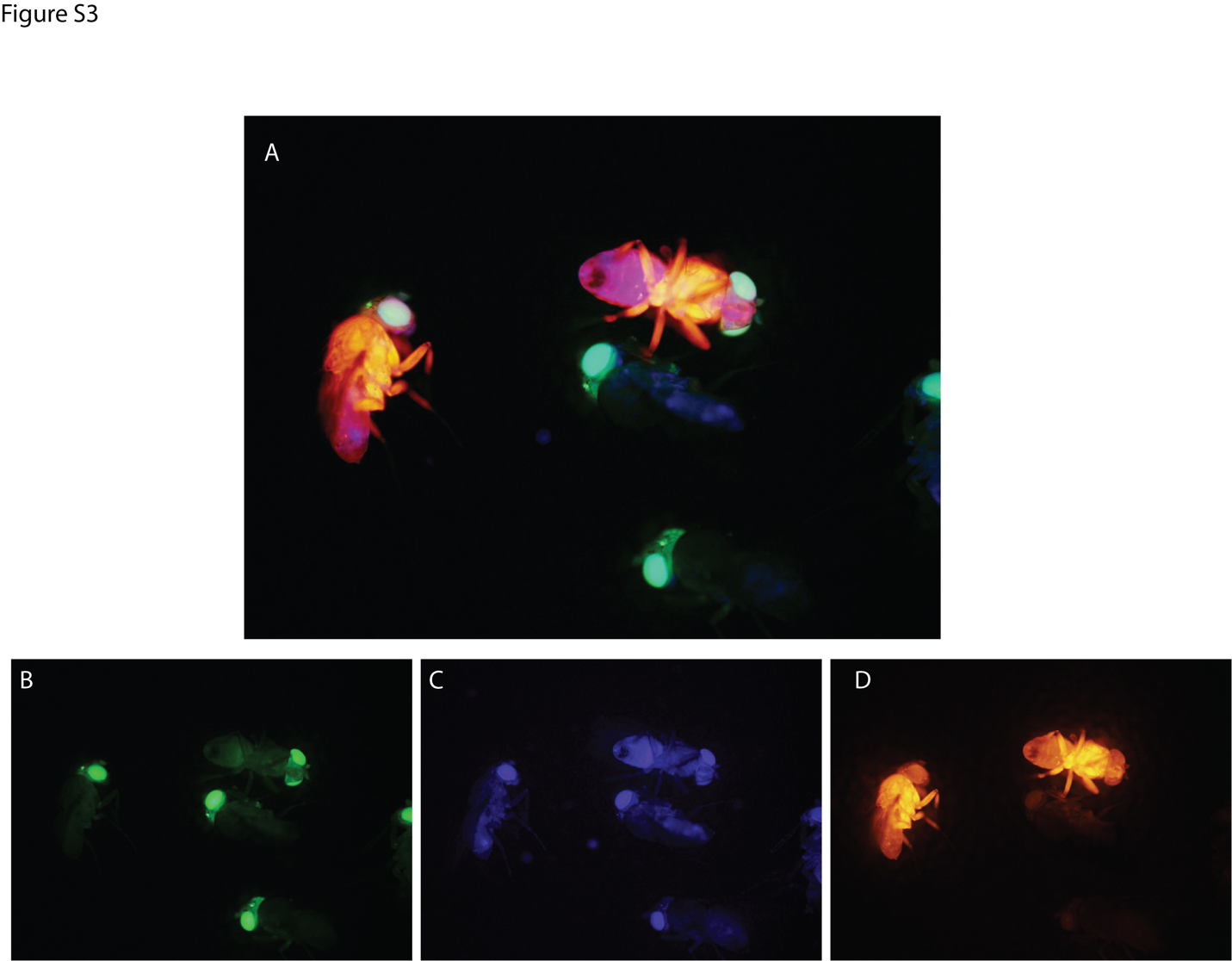


Figure S3 – Example of flies segregating for three transgenes driving fluorescent proteins in two anatomical domains. (A) Flies expressing 3XP3-EYFP and 3XP3-mTurquoise and segregating for MHC-687-DsRed. (B-D) The three channels, green (B), blue (C), and red (D) illustrate that the colors and anatomical patterns can be clearly distinguished.
